# Protein Structure Refinement Using Multi-Objective Particle Swarm Optimization with Decomposition Strategy

**DOI:** 10.3390/ijms22094408

**Published:** 2021-04-23

**Authors:** Cheng-Peng Zhou, Di Wang, Xiaoyong Pan, Hong-Bin Shen

**Affiliations:** 1 Institute of Image Processing and Pattern Recognition, Shanghai Jiao Tong University, and Key Laboratory of System Control and Information Processing, Ministry of Education of China, Shanghai 200240, China; anakinchou@sjtu.edu.cn (C.-P.Z.); wangdi19941224@sjtu.edu.cn (D.W.); 2008xypan@sjtu.edu.cn (X.P.); 2 Department of Computer Science, Shanghai Jiao Tong University, Shanghai 200240, China

**Keywords:** protein structure prediction, structure refinement, multi-objective particle swarm optimization, decomposition strategy, AIR

## Abstract

Protein structure refinement is a crucial step for more accurate protein structure predictions. Most existing approaches treat it as an energy minimization problem to intuitively improve the quality of initial models by searching for structures with lower energy. Considering that a single energy function could not reflect the accurate energy landscape of all the proteins, our previous AIR 1.0 pipeline uses multiple energy functions to realize a multi-objectives particle swarm optimization-based model refinement. It is expected to provide a general balanced conformation search protocol guided from different energy evaluations. However, AIR 1.0 solves the multi-objective optimization problem as a whole, which could not result in good solution diversity and convergence on some targets. In this study, we report a decomposition-based method AIR 2.0, which is an updated version of AIR, for protein structure refinement. AIR 2.0 decomposes a multi-objective optimization problem into a number of subproblems and optimizes them simultaneously using particle swarm optimization algorithm. The solutions yielded by AIR 2.0 show better convergence and diversity compared to its previous version, which increases the possibilities of digging out better structure conformations. The experimental results on CASP13 refinement benchmark targets and blind tests in CASP 14 demonstrate the efficacy of AIR 2.0.

## 1. Introduction

The functions of a protein are closely related to its 3D structure, and high-resolution protein structure can increase the understanding of what it does and how it works. In the past decades, dramatic progress has been made in structure determination using wet-lab experimental methods, such as X-ray crystallography, nuclear magnetic resonance (NMR) spectroscopy, and recent electron microscopy techniques [1]. However, these experiments are still expensive and time-consuming [2]. Many popular automated protein structure prediction methods play important complementary roles [3,4,5], such as AlphaFold [6], trRosetta [7], I-TASSER [8], and MULTICOM [9,10]. Especially in recent years, protein structure prediction performance has been largely improved due to the advances in both theoretical and computational studies as demonstrated in recent CASP (Critical Assessment of protein Structure Prediction) assessment, e.g., coevolution analysis-based investigation [11,12,13,14], powerful deep learning computational techniques [15,16,17], etc.

Although remarkable results have been achieved in protein structure prediction, the predicted models still contain inaccurate regions deviating from the native structures [18]. Thus, there have been increasing efforts on improving predicted models via refinement as a following step. Since the 8th competition of Critical Assessment of protein Structure Prediction, the protein structure refinement task has been introduced to evaluate the performance of computational methods for structure refinement by given an initial predicted model [19,20,21]. However, it is a challenging task until now, as it is a blind refinement and on some hard targets, refinement methods degrade their initial models rather than improve them.

One of the common strategies for protein structure refinement is to implement the work pipeline through the combination of energy functions and optimization algorithms [22,23,24,25]. The energy function is designed to describe a protein’s state that is near-native or non-native from its view, which will guide the refinement search to its lower energy state. Considering its importance, a number of molecular mechanics force fields and knowledge-based energy functions have been proposed, i.e., AMBER [26], CHARMM [27], OPLS [28], RWplus [29], DFIRE [30], GOAP [31], and Rosetta [32]. However, it is still difficult to apply a single energy function to exactly describe the states of all proteins due to the large diversities of the protein structures. Each energy function would have its advantages and disadvantages on specific targets, which is a potential reason the performance of the refinement algorithms often varies with the targets in the CASP experiments.

In addition to the energy functions, the optimization algorithms are also crucial in protein structure refinement, which are designed to search for the lowest-energy structure conformation. Popular optimization algorithms include Molecular Dynamics (MD) simulation [33] and Monte Carlo (MC) simulation [34]. It is still very challenging to achieve consistent refinement over initial models, and one potential reason is that most existing approaches are conducted based on a single energy function. 

Motivated by those observations, we have developed one multi-objective-based refinement method called AIR [35] to alleviate the potential bias caused by minimizing only one energy function. The AIR is a multi-objective particle swarm optimization (PSO)-based protocol [36], where each structure is treated as a particle. The quality of the particles in each iteration is evaluated by three energy functions based on dominance relations [37], and the non-dominated particles are put into a set called Pareto set (PS) [38], which is used to select the final refined structures.

However, the dominance-based AIR has no direct control over the movement of each particle in the swarm and no suitable mechanism to maintain the diversity of Pareto front (PF) [39]. The loss of diversity may deteriorate the advantage of multi-objective optimization. Moreover, the crucial parameter Gbest in PSO is difficult to choose, since there are many non-dominated candidates in the PS. Using Pareto dominance alone would deteriorate the selection pressure toward the PF and slow down the searching process [40], since the update of another important parameter Pbest only needs to reduce one energy function. 

To solve the above problems, we present a decomposition-based approach AIR 2.0, which is an updated version of AIR 1.0 to further increase the conformation optimization capability. In AIR 2.0, each particle is associated with a unique subproblem defined by a weight vector, which is different from the protocol of AIR 1.0 that solves a multi-objective optimization problem as a whole. Thus, the diversity is accordingly improved, since each particle is moving toward PF in its own direction. In addition, the Pbest and Gbest of each particle have a determined choice according to its own subproblem, helping avoid oscillation in the searching process. The benchmark tests of CASP13 refinement targets and blind tests in CASP14 demonstrate the efficacy of the new updated version of AIR refinement pipeline.

## 2. Experiments and Results

We have evaluated AIR 2.0 pipeline on the refinement targets of CASP13 and CASP14. To demonstrate the advantage of AIR 2.0, we compare it with state-of-the-art methods, including its previous version AIR 1.0 and other state-of-the-art refinement methods in CASP13 such as FEIGLAB [18], BAKER [24], and Zhang-Refinement [41]. Global distance test total score (GDT-TS) [42], template modeling score (TM-score) [43], and root mean square deviation (RMSD) are the metrics for evaluating the effectiveness of AIR 2.0. 

For each target, the number of divisions H in (10) (see Methods) is set to 10 according to our local tests, resulting in $N=\left(\begin{array}{c} H+M-1\\ M-1 \end{array}\right)=\left(\begin{array}{c} 10+3-1\\ 3-1 \end{array}\right)=66$ weight vectors or subproblems and the same number of particles. M=3 is the number of objectives. The single initial model provided by CASP is taken as input, and another 65 models are generated by applying random perturbations to the initial model. The neighborhood size T is set to 8 according to [44], and the maximum number of iterations is set to 3000 as AIR 1.0. We set S = 20,000 in (9) (see Methods) to get a stable result and output the top five ranked solutions. 

### 2.1. Effectiveness of AIR 2.0 on CASP13

**Figure 1:**
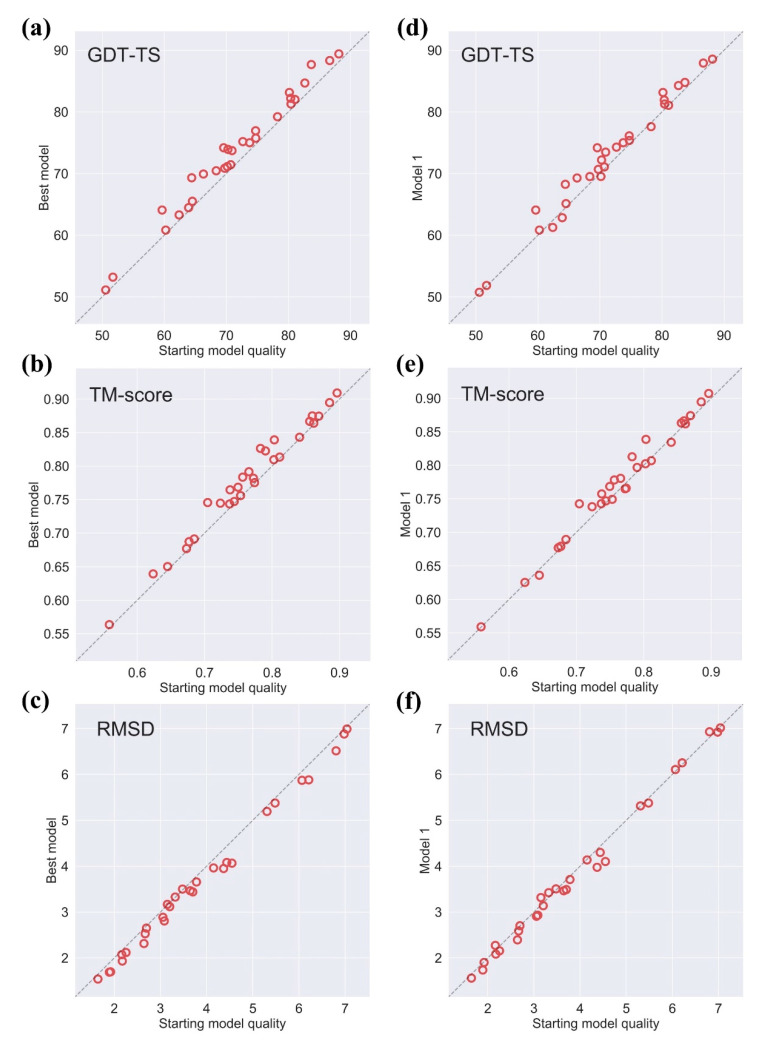
Effectiveness of AIR 2.0 on CASP13 measured by GDT-TS, TM-score, and RMSD. The comparison of the best model refined by AIR 2.0 and the initial model in terms of GDT-TS, TM-score, and RMSD are shown in (**a**–**c**), respectively. The comparison of AIR 2.0 refined Model 1 and the initial model in terms of GDT-TS, TM-score, and RMSD are given in (**d**–**f**) respectively.

We test AIR 2.0 on the 29 CASP13 refinement targets (two cancelled targets were excluded), and the results are summarized in Figure 1. We compare the best model and Model 1 with the initial model. The best model achieves consistent improvements over the initial model and almost all targets are to a certain degree refined. The average gains in the quality of the best model are +1.98 in GDT-TS, +0.014 in TM-score, and −0.18 Å in RMSD. Compared to Model 1, the average improvement in GDT-TS is 1.22, and 82% of the targets (24 out of 29 targets) are refined. In terms of TM-score and RMSD, the average improvements are +0.0076 and −0.0752 Å with 72% (21 out of 29 targets) and 69% (20 out of 29 targets) being refined, respectively.

**Figure 2:**
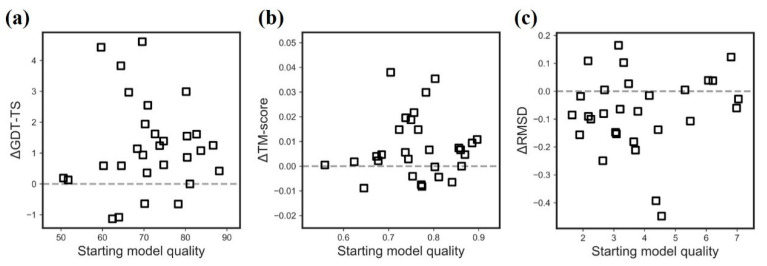
Refinement improvement over the initial model in terms of (**a**) GDT-TS (**b**) TM-score (**c**) RMSD.

It is observed that the targets with a medium quality are more likely to be refined (Figure 2). Specifically, AIR 2.0 improves those targets with the following quality: (1) initial GDT-TS is between 60 and 80, (2) initial RMSD is between 2 and 5 Å, and (3) initial TM-score is between 0.65 and 0.8. The potential reason is that high quality models leave a few spaces to refine, while the relatively bad models might be trapped in a deep local minimum caused by a rough energy landscape.

### 2.2. AIR 2.0 Is Superior to AIR 1.0

**Figure 3:**
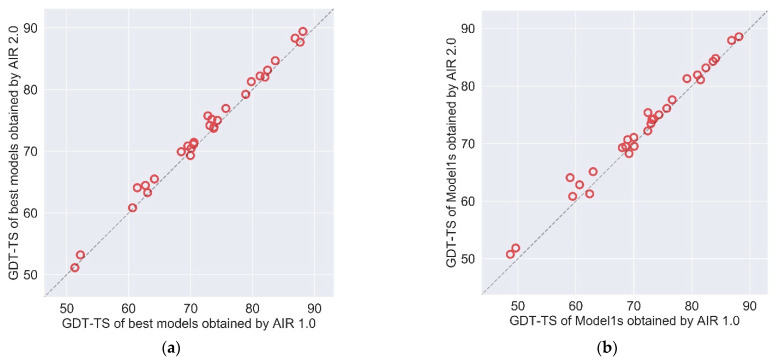
The GDT-TS comparison of (**a**) best models and (**b**) Model1s between AIR 1.0 and AIR 2.0 on the targets of CASP13.

We compare the updated method AIR 2.0 with our previous AIR 1.0. The CASP13 results of AIR 2.0 and AIR 1.0 are summarized in Table 1. Figure 3 illustrates the GDT-TS of all targets refined by AIR 2.0 and 1.0 based on the best model and Model 1. The results indicate that AIR 2.0 achieves better or comparable performance over AIR 1.0. Compared to AIR 1.0, AIR 2.0 obtains a better quality in 21 out of 29 targets for the best model and 24 of 29 targets for Model 1. 

**Figure 4:**
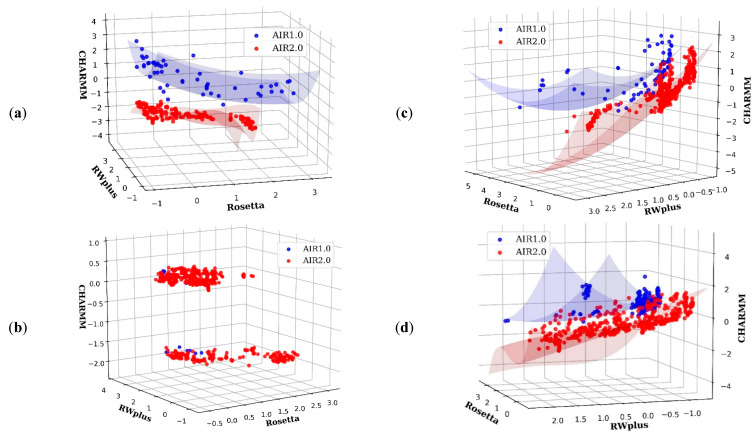
Pareto fronts of some targets obtained by AIR 2.0 (red) and AIR 1.0 (blue). (**a**) R0976D2, (**b**) R0981D5, (**c**) R0999D3, and (**d**) R1001. AIR 2.0 finds more non-dominated solutions than AIR 1.0, and these solutions are distributed with a high diversity on the target (**b**). Moreover, for targets such as (**a**,**c**,**d**), the solutions obtained by AIR 2.0 completely dominate those obtained by AIR 1.0, indicating a more convergence toward the true Pareto front.

For AIR 1.0, a refinement of hard targets in CASP13 often obtained model degradation rather than improvement, such as R0949, R0977D2, R0996D4, and R1016. However, AIR 2.0 achieves promising refinement results on these hard targets. The potential reason is due to the diversity of PS introduced by the decomposition strategy. In the case of R0981D5, the non-dominance solutions in the final PS of AIR 2.0 and 1.0 are plotted in Figure 4b. We can see that AIR 2.0 finds more non-dominated solutions than AIR 1.0, and these solutions are distributed with a high diversity. Moreover, as shown in Figure 4a,c,d, the solutions obtained by AIR 2.0 completely dominate those obtained by AIR 1.0, indicating a more convergence toward the true PF. Thus, the overall quality of the solutions obtained by AIR 2.0 is higher than those of AIR 1.0, which is beneficial for the selection of high quality Model 1.

The dominance-based method AIR 1.0 drives the whole population toward the PF without direct control over the movement of each individual in the population. Thus, AIR 1.0 prefers the regions that are easy to access and does not sufficiently account for the diversity. As a result, the solutions obtained by AIR 1.0 are only distributed in a small area. Moreover, due to the lack of stable guidance on Pbest and Gbest (see Methods), the searching process of each particle would be difficult. However, the decomposition strategy in AIR 2.0 assigns a single objective optimization subproblem for each particle. In this way, each particle has an exact update direction or a clear target position in PF, which results in better diversity and convergence features. This is the potential reason why AIR 2.0 outperforms AIR 1.0 in most targets. 

### 2.3. Comparison with Other State-of-the-Art Refinement Methods

The test data consist of those targets in which each group performs the best on CASP13 in order to highlight the characteristics of each method. The refined models of BAKER, FEIGLAB, and Zhang are available at the CASP official website. As shown in Table 2, AIR 2.0 yields promising improvement over initial models. For the target R0949, R0979, and R0989D1, AIR 2.0 is the only method that achieves improvement rather than degradation over initial models and the GDT-TS gains is 0.59, 3.53, and 0.37, respectively. However, for the targets such as R0968s1, R0974s1, and R0986s1, the GDT-TS gains obtained by BAKER or FEIGLAB are larger than AIR 2.0. The potential reason is probably that AIR 2.0 uses multiple energy functions that constrain each other, resulting in a limited deviation from initial models. These results also highlight that the protein structure map is huge, and it is very hard to achieve a general better refinement algorithm on all proteins. For hard targets, AIR 2.0 would be reliable, since we extend the one-dimension optimization to a new three-dimension space optimization, partially alleviating the bias caused by using only one energy function.

### 2.4. Blind Test in CASP14

**Figure 5:**
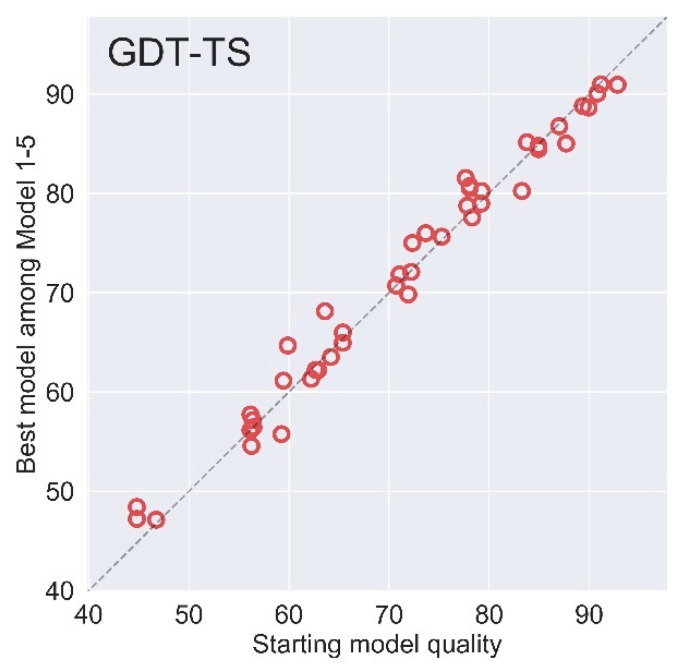
The overall performance on 44 refinement targets of AIR in CASP14 blind test measured by GDT-TS.

We also test our method in the recent CASP14 blind test. Overall, our AIR ranks the ninth among 37 groups in the competition according to SUM Zscore > −2.0 (including the reference group named STARTING MODEL). There are 51 refinement targets in total, where two targets were cancelled during the competition and five targets do not have a native structure for reference. The results of AIR on the remaining 44 targets are summarized in Figure 5 (for more details, please refer to the CASP14 website). The average gains in the quality of the best model among Model 1–5 are +0.36 in GDT-TS, which is slightly lower than CASP13. The main reason is that our solution ranking method performed relatively poorly on these targets, and we found locally that a number of better structures were not selected as the top five submission models. This is also one of our future efforts to further improve the AIR program. However, there are also some successful cases. For example, AIR ranks the first on the target R1042v2 among the models submitted by 31 groups (https://predictioncenter.org/casp14/results.cgi?view=tables&target=R1042v2&model=1&groups_id=, accessed on 21 April 2021). Considering the Model 1 submission models, the AIR approach is the only one that successfully achieves improvement rather than degradation over the initial model. Considering the best submission models, our predictions for the target R1029 are among the most accurate in all submissions (https://predictioncenter.org/casp14/results.cgi?view=tables&target=R1029&model=all&groups_id=, accessed on 21 April 2021). 

The success of these two cases indicates the potential advantages of multi-objective optimization and the PSO algorithm that can efficiently explore the high-dimensional energy landscape to get a reliable refined model. On the other hand, the performance of AIR in CASP14 also indicates that there is still room for improvement of our algorithm. For instance, on the target R1042v2, the improvement is still limited to a moderate level. For target R1029, our Model 2 submission is better than our Model 1, implying that we still need to investigate how to rank the final solution. In our AIR’s future development, we will go on to find a better mechanism that could guide the search process to achieve significant improvement and a new method to accurately score all the candidate solutions.

## 3. Discussion

### 3.1. The Importance of the Diversity on AIR 2.0

In AIR 1.0, we have shown that multi-objective optimization is a promising way to improve protein structure refinement. The two goals of the multi-objective optimization are: (1) a set of solutions as close as possible to the PF; (2) a broadly distributed set of solutions that cover the entire PF [45]. The two goals are also referred to convergence and diversity. In the field of protein structure refinement, the diversity of the solutions is important. When given a model to be refined, we have no idea which direction or what combination of multiple energy functions is feasible for improvement due to the diversity of protein structures. In order to improve the initial model, AIR 2.0 tries different directions to obtain a well-distributed PF that covers all potential solutions. As mentioned before, dominance-based method AIR 1.0 prefers the regions that are easy to access, resulting in insufficient diversity, which may lose the solution diversity. 

**Figure 6:**
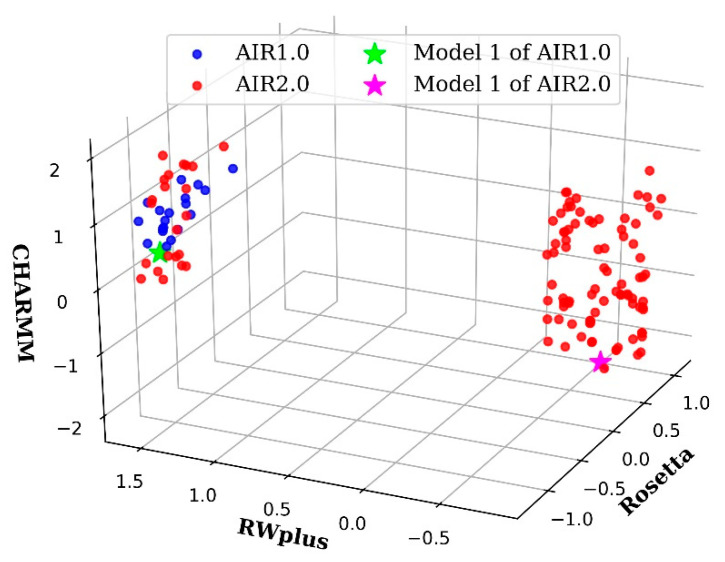
Candidate solutions obtained by AIR 2.0 (red) and AIR 1.0 (blue) on the target R0949. The Pareto front of R0949 is irregular and consists of at least two parts. The solutions of AIR 1.0 covers only one part, and the GDT-TS of its Model 1 marked with a green star is 62.98, which is a degradation to the initial model with a GDT-TS of 64.53. However, Model 1 of AIR 2.0 marked with a magenta star yields a GDT-TS of 65.12 on the other part of the Pareto front, demonstrating improvement over the initial model.

To give a more intuitive understanding of the conformation solution diversity, Figure 6 shows a comparison between AIR 1.0 and AIR 2.0 on the target R0949 from CASP13 whose PF is irregular, consisting of at least two parts. The solutions of AIR 1.0 cover only one part, and the GDT-TS of the Model 1 is 62.98, which is a degradation of the initial model with a GDT-TS of 64.53. However, Model 1 of AIR 2.0 yields a GDT-TS of 65.12 on the other part of the PF, demonstrating improvement over the initial model. Therefore, if we do not take the diversity carefully, the possibility of improvement would decrease.

### 3.2. The Influence of Hyperparameters on AIR 2.0 

The neighborhood size T (see Methods) is a major control parameter in AIR 2.0 since the solutions in the neighborhood of a subproblem can be used to guide the searching process. In a sense, each subproblem with its neighborhood is regarded as a swarm. To test the influence of T, we perform some experiments on those targets with different size. The results are summarized in Table 3. When T=3, the neighborhood is too small, and the particles in a swarm are similar, resulting in the inability to explore the new area and achieve a good result. It should also be noted that AIR 2.0 performs similarly with T larger than 8. That is because the gbest for each subproblem depends on only a certain number of neighbors, while others play a small role. Moreover, a large T will increase the computational burden and might undermine the diversity of solutions. Thus, we set T=8 to make a tradeoff between the performance and running time.

The penalty value θ (see Methods) in the PBI decomposition approach is another important parameter [46]. In this study, we adopt an adaptive penalty scheme (APS) [47] that linearly increases θ with the number of generations from 5 to 20. At the early stage, a small θ is beneficial for convergence toward *PF*, and the value of θ is gradually increased to promote the diversity of solutions. For the number of iterations MaxIt and the number of particles N, generally, the larger the two numbers are, the better the performance is. However, large values of these two parameters will increase the time cost. To make a tradeoff, we finally set MaxIt=3000 and N=66.

## 4. Methods

### 4.1. Overview of Refinement Pipeline AIR 2.0

**Figure 7:**
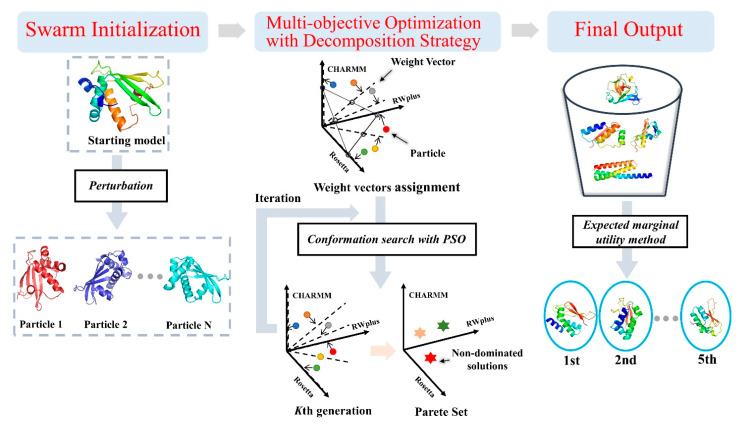
Overall refinement protocol of AIR 2.0. The protocol consists of swarm initialization, multi-objective optimization with decomposition strategy, and solution ranking. In the second step, the different colored circles and stars denote particles and non-dominated solutions respectively.

As shown in Figure 7, AIR 2.0 consists of three main steps. The first step is swarm initialization, which generates multiple particles. If a single initial model is used as the input, other particle models can be generated by applying perturbations to the initial one. In total, an initial swarm of N particles is obtained. In the second step, each particle is associated with a unique weight vector generated by the simplex-lattice design method [48]. Then, the main loop of optimization is performed, where Rosetta, RWplus, and CHARMM are selected as three fitness functions. Each particle updates the position according to its own subproblem formulated by the weight vector. In each iteration, the non-dominated solutions in the whole population are added to the Pareto set. In the third step, all solutions in the Pareto set are ranked using the expected utility rule [49] and the top five of them are selected as the final refined structures.

### 4.2. Representations of Protein Conformations

Mathematically, protein conformation could be represented by the Cartesian coordinates of the atoms or internal coordinates (bond lengths and angles) [50]. The former is suitable for describing physical force fields, and the latter is a better representation to describe bonded interactions as well as certain kinds of experimental information [51]. In AIR 2.0, we use both coordinate systems.

During the sampling stage, the protein backbone is represented by a list of main-chain torsion angles using internal coordinates:

(1) $$C=\left[\phi_{1},\psi_{1},\omega_{1},\ldots ,\phi_{L},\psi_{L}\right]$$

where L stands for the protein length. We further use the Denavit and Hartenberg (DH) method [52] to convert internal coordinates to corresponding Cartesian coordinates, since certain energy functions, such as the Rosetta, explicitly encode Cartesian energy terms. This conversion goes back and forth until the end of the pipeline. 

### 4.3. Multi-Objective Optimization

Similar to its previous version, the AIR 2.0 uses three energy functions to perform conformation search in a 3D energy space composed by Rosetta, RWplus, and CHARMM. It is crucial to select accurate force fields for protein structure refinement. There are roughly two types of force fields in the community. One is physics-based force fields that are designed on the basis of all kinds of interactions at the atomic and molecular levels. The other is knowledge-based energy function deduced from diverse sets of known protein structures. Each type of force field has it its merits and drawbacks. To take advantage of both types of force fields, we choose one popular physics-based force field CHARMM and one typical knowledge-based energy function RWplus. Rosetta energy function could be classified into both types and is widely used in protein structure prediction and refinement for its good performance. Therefore, we use it as a complementary part for the other two force fields. This will formulate the protein structure refinement as a multi-objective optimization (MOP) problem as follows:

(2) $$minimize\;F\left(C\right)={\left(f_{Rosetta}\left(C\right),f_{RWplus}\left(C\right),f_{CHARMM}\left(C\right)\right)}^{T}\;subject\;to\;C\in \Omega \;\;$$

where C is the conformation of a protein and Ω is the overall conformational space. $f_{Rosetta}\left(C\right)$, $f_{RWplus}\left(C\right)$, and $f_{CHARMM}\left(C\right)$ are three energy values in terms of C.

Due to potential conflicts among multiple objectives, usually, one single solution (conformation) cannot optimize all objectives simultaneously. Instead, a set of optimal solutions representing the trade-offs among different objectives could be obtained. A dominance relation between different solutions is often used to suggest the acceptance of current conformations. 

Let $C_{i},C_{j}\in \Omega$; we say that $C_{i}\;dominates\;C_{j}\;$ (denoted as $C_{i}<C_{j}$) if and only if $\forall k=1,2,3,f_{k}\left(C_{i}\right)\leq f_{k}\left(C_{j}\right)$ and $F\left(C_{i}\right)\neq F\left(C_{j}\right)$, where $f_{1}$,$\;f_{2}$, and $f_{3}$ correspond to $f_{Rosetta}\left(C\right)$,$\;f_{RWplus}\left(C\right)$, and $f_{CHARMM}\left(C\right)$ respectively. If $C^{*}\in \Omega$ and there is no other solution in Ω that dominates $C^{*}$, then $C^{*}$ is considered as a Pareto optimal solution. The Pareto set (PS) is defined as:

(3) PS={C∈Ω|C is a Pareto optimal solution}.

The energy map of all Pareto optimal solutions in PS is called a Pareto front (PF) [53] and can be described as:

(4) $$PF=\left\{F\left(C\right)={\left(f_{Rosetta}\left(C\right),f_{RWplus}\left(C\right),f_{CHARMM}\left(C\right)\right)}^{T}|C\in PS\right\}.$$

The goal of multi-objective optimization is to obtain widely distributed Pareto optimal solutions that are as close to true PF as possible.

### 4.4. Decomposition Approach in Multi-Objective Optimization

Our original protocol AIR 1.0 solves the multi-objective optimization based on Pareto dominance [37]. It mainly evaluates each solution by its Pareto dominance relations to other solutions and aims to drive the population toward the PF as a whole. However, the movement of each particle in the population and the distribution of computational effort over different ranges of the PF can be further investigated. Otherwise, the whole population would prefer to the regions that are easily accessible and cannot maintain the diversity of the solutions.

Generally speaking, a Pareto optimal solution for the multi-objective optimization problem can be seen as the optimal solution of a scalar optimization subproblem whose objective is an aggregation function of all the individual objectives ($f_{Rosetta}$,$\;f_{RWplus}$, $f_{CHARMM}$) [44]. Thus, a multi-objective optimization problem can be decomposed into a number of optimization subproblems, and each subproblem is distinguished by one unique weight vector. Then, Pareto solutions could be achieved by minimizing such subproblems. There exists several methods to construct the aggregation function [54] for each subproblem with a weight vector, such as weight sum approach [44], Tchebycheff approach [55], penalty-based boundary intersection (PBI) [44], etc. Here, AIR 2.0 uses the PBI approach to construct the aggregation function for each subproblem. Formally, an optimization subproblem in AIR 2.0 can be stated as:

(5) $$minimize\;g^{pbi}\left(C|\lambda_{i},z^{*}\right)=d_{1}+\theta d_{2}\;subject\;to\;C\in \Omega$$

where

(6) $$d_{1}=\frac{∥{\left(F\left(C\right)-z^{*}\right)}^{T}\lambda_{i}∥}{∥\lambda ∥}$$

(7) $$d_{2}=∥F\left(C\right)-\left(z^{*}+d_{1}\frac{\lambda_{i}}{∥\lambda_{i}∥}\right)∥$$

where *C* is a candidate solution (conformation) that belongs to overall conformational space. $F\left(C\right)={\left(f_{Rosetta}\left(C\right),f_{RWplus}\left(C\right),f_{CHARMM}\left(C\right)\right)}^{T}$ consists of three components from Rosetta, RWplus, and CHARMM. $\lambda_{i}={\left(\lambda_{i}^{1},\lambda_{i}^{2},\lambda_{i}^{3}\right)}^{T}$ is the weight vector of the ith subproblem satisfying $\lambda_{i}^{j}\geq 0$ and $\sum_{j=1}^{3}\lambda_{i}^{j}=1$. $z^{*}={\left(z_{Rosetta}^{*},z_{RWplus},z_{CHARMM}^{*}\right)}^{T}$ is the ideal objective vector with $z_{k}^{*}\leq \underset{C\in \Omega }{\min}f_{k}\left(C\right),\;k\in \left\{Rosetta,RWplus,CHARMM\right\},\;\theta$ is a user-defined penalty parameter. $d_{1}$ is the distance between the ideal objective vector $z^{*}$ and the solution F(C), $d_{2}$ is the direction error between $\lambda_{i}$ and *F(C)*. The PBI approach tries to minimize both $d_{1}$ and $d_{2}$, and their relative importance is control by θ. 

**Figure 8:**
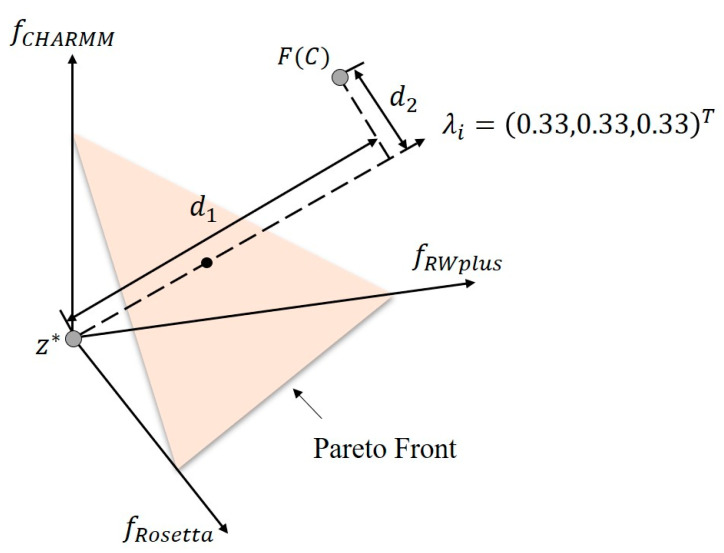
Illustration of PBI approach with a weight vector $\lambda_{i}={\left(0.33,\;0.33,0.33\right)}^{T}$. *F(C)* is the corresponding point of the candidate solution in the energy space. $z^{*}$ is the ideal objective vector. $d_{1}$ is the distance between the ideal objective vector $z^{*}$ and the solution F(C), *d*_2_ is the direction error between *λ_i_* and *F(C)*. The Pareto front is represented by an orange plane. The PBI approach tries to minimize both *d*_1_ and *d*_2_. Obviously, the intersection of the weight vector and the Pareto front marked by a black point is the optimal solution of the subproblem defined by PBI with *λ_i_*. It should be noted that the Pareto front is always irregular and discontinuous in practice. Here, we use a simple plane to represent it just for clarity.

Figure 8 presents a simple example to illustrate the PBI approach given the weight vector $\lambda_{i}={\left(0.33,\;0.33,0.33\right)}^{T}$. *F(C)* and $z^{*}$ are denoted as two points in the energy map. The orange plane is PF, and $d_{1}$, $d_{2}$ are marked in the figure. It is clear that the intersection of the weight vector and PF, which is marked by a black point, is the optimal solution of the subproblem defined by PBI with $\lambda_{i}\;$.

Thus, the optimal solution to (5) is a Pareto optimal solution to (2). We use $\lambda_{i}$ to emphasize that (5) is the ith subproblem defined by a weight vector. In order to obtain a set of different Pareto optimal solutions, we can use different weight vectors. A natural idea comes that if we have a large number of uniformly distributed weight vectors, we could get a set of Pareto optimal solutions that approximates PF very well.

### 4.5. Particle Swarm Optimization

Particle swarm optimization (PSO) [56] is a meta-heuristic algorithm simulating the behaviors of groups of birds and fishes. It solves a problem by iteratively improving candidate solutions with the information coming from their population. Each candidate solution not only has its own exploration behavior, but its trajectory is also affected by other solutions in the population. In PSO, every individual in the population is called a particle, and swarm is another name for population. In AIR 2.0, each particle in the swarm represents a candidate conformation in the overall conformational space. A particle is characterized by its position and velocity, where the position is the conformation of a protein represented by (1) and the velocity represents the change of torsion angles. The particle uses the position of the selected global leader and its own personal movement trajectory to update the velocity and position values using (8) and (9).

(8) $$v_{i}^{t+1}=w*v_{i}^{t}+c_{1}*r_{1}*\left(Pbest_{i}^{t}-C_{i}^{t}\right)+c_{2}*r_{2}*\left(Gbest_{i}^{t}-C_{i}^{t}\right)$$

(9) $$C_{i}^{t+1}=C_{i}^{t}+v_{i}^{t+1}$$

where $v_{i}^{t}$ is the velocity of the ith particle in the tth generation, $C_{i}^{t}$ is the new conformation of the ith particle in the tth generation, and w is the inertia weight. 

According to our previous study [35], we set w to 1.3 at the beginning; it linearly decreases to 0.7 as the number of iterations increases.$\;c_{1}$ and$\;c_{2}$ are two learning coefficients that are both set to be 2 [57].$\;r_{1}$,$r_{2}\in \left[0,1\right]$ are uniformly distributed random variables. $Pbest_{i}^{t}$ is the best conformation that the ith particle has ever been until the tth generation. Similarly, $Gbest_{i}^{t}$ is the best conformation that the whole swarm has ever met until the tth generation. Each time the conformation updates, the non-dominated ones are added into PS.

For only one objective, each solution can be ranked according to the objective. Thus, both $Pbest_{i}^{t}$ and $Gbest_{i}^{t}$ have a determined choice. However, for multiple objectives, there are always many non-dominated solutions that are equally good under the concept of the dominance. Thus, it is difficult to choose $Pbest_{i}^{t}$ and $Gbest_{i}^{t}$ to lead the searching process. In our previous protocol of AIR 1.0, $Pbest_{i}^{t}$ is updated when any one of the three energy functions decreases, and $Gbest_{i}^{t}$ is randomly selected from current PS. This will severely deteriorate the selection pressure toward the *PF* and considerably slows down the searching process due to ambiguous search direction. 

### 4.6. Obtaining Pareto Optimal Set with Multi-Objective Particle Swarm Optimization Based on Decomposition Strategy

Due to the diversity of protein structures, we use multiple energy functions as multi-objectives to alleviate the bias problem caused by minimizing one single energy function. Given a particular protein, which energy function or what combination of these energy functions is appropriate for a particular protein is still unknown. Each candidate solution on PF represents a potential optimization direction. Thus, the diversity of solutions on the PF is of importance for multi-objective optimizations in protein structure refinement. In order to make good use of three energy functions, we need to find as many Pareto optimal solutions as possible and maximize the distribution of solutions in the PF. However, using Pareto dominance alone could discourage the diversity of solutions, since it has no direct control over the movement of each individual in its population and no good mechanism to control the distribution of the computational effort over different ranges of the PF. As a result, the whole population is updated in a random direction and prefers those regions that are easily accessible. Finally, the solutions will end up in a small range of PF, resulting in the loss of the diversity.

In order to overcome the above shortcomings, AIR 2.0 uses a decomposition strategy to define a single objective optimization subproblem for each particle. A Pareto optimal solution to an MOP could be an optimal solution of a scalar optimization subproblem, in which the objective is an aggregation of all the objectives in AIR 2.0. In this way, each particle has an exact updating direction and increasing evolutionary pressure, which is beneficial to the convergence. In addition, the diversity is inherently guaranteed since each particle is moving toward PF in its own direction. The general framework is as follows.

At the beginning, a set of weight vectors $\left\{\lambda_{1},\ldots ,\lambda_{N}\right\}$ (N is the number of particles) are generated using the canonical simplex-lattice design method [48], whose weight vectors are sampled from a unit simplex.

(10) $$\{\begin{array}{c} \lambda_{i}=\left(\lambda_{i}^{1},\lambda_{i}^{2},\lambda_{i}^{3}\right)\;\\ \lambda_{i}^{j}\in \left\{\frac{0}{H},\frac{1}{H},\ldots ,\frac{H}{H}\right\},\overset{3}{\underset{j=1}{\sum }}\lambda_{i}^{j}=1 \end{array}$$

where i=1,…,N is the index of uniformly distributed weight vector. $\lambda_{i}$ has three components corresponding to three energy functions, Rosetta, RWplus, and CAHRMM. H>0 is the number of divisions along each objective coordinate. In total, there are $N=\left(\begin{array}{c} H+M-1\\ M-1 \end{array}\right)=\left(\begin{array}{c} H+3-1\\ 3-1 \end{array}\right)$ different weight vectors for M=3 objectives. Then, each particle is associated with a different weight vector, which defines a unique subproblem. Solving these subproblems is equivalent to solving the original multi-objective optimization problem.

In AIR 1.0, the velocity and position of a particle are updated using the information from its individual best $Pbest_{i}^{t}$ and the global best $Gbest_{i}^{t}$. However, it is difficult to select a suitable one, since multiple objectives result in a large number of equally good non-dominated solutions. Now with the decomposition strategy, $Pbest_{i}^{t}$ is obvious for a particle with a weight vector $\lambda_{i}$ using the aggregation function $g^{PBI}\left(C|\lambda^{i},z^{*}\right)$. For $Gbest_{i}^{t}$, there is a small difference, since each particle corresponds to a different subproblem. However, if two weight vectors $\lambda_{i}$ and $\lambda_{j}$ are close enough, the optimal solutions to both two subproblems, $g^{PBI}\left(C|\lambda_{i},z^{*}\right)$ and $g^{PBI}\left(C|\lambda_{j},z^{*}\right)$, will also be similar. Therefore, the information from the searching process of *λ_i_* is useful to $\lambda_{j}$ and vice versa. According to this observation, a neighborhood of the weight vector $\lambda_{i}\;$is defined as a set of weight vectors $\left\{\lambda_{i_{1}},\lambda_{i_{2}}\ldots ,\lambda_{i_{T}}\right\}$ that are closest to $\lambda_{i}$, where T is the size of the neighborhood. Correspondingly, the neighborhood of the ith particle is composed of those particles whose weight vectors are in the neighborhood of $\lambda_{i}$. With the notion of neighborhood, $Gbest_{i}^{t}$ is defined as the best position in the neighborhood of the ith particle during t generations. Then, we could use the particle swarm optimization algorithm to optimize those subproblems simultaneously and finally obtain a Pareto optimal set. The pseudocode of the main framework for AIR 2.0 is summarized in Algorithm 1.
**Algorithm 1** Main Framework of AIR 2.0**Input:** Initial model $C_{0}$, the maximum number of iterations *MaxIT*, the number of particles *N*.**Output:** Pareto set PS.   /*Initialization*/
Generate weight vectors $\left\{\lambda_{1},\ldots ,\lambda_{N}\right\}$ using the simplex lattice method.Create initial population $\left\{C_{1}^{0},\ldots ,C_{N}^{t}\right\}$ by perturbing $C_{0}$ and assign weight vectors to each particle individually.Compute the Euclidean distances between any two weight vectors. For each particle $C_{i}^{0}$, i=1,…,N, set $B\left(i\right)=\left\{i_{1},..i_{T}\right\}$, where $\lambda_{i_{1}},\ldots ,\lambda_{i_{T}}$ are the *T* closest weight vectors to $\lambda_{i}$.Initialize ideal objective vector $z^{*}$, set the initial velocity randomly, $pbest_{i}^{0}=gbest_{i}^{0}=C_{i}^{0}$, add initial non-dominated particles into PS.   /*Main Loop*/**while**t<MaxIt**do:****for**i=1,…,N**do:**Update the position of the $C_{i}^{t}$ using PSO Formulas (8) and (9).Update $z^{*}$**if**$g\left(C_{i\;}^{t}|\lambda_{i}\right)<g\left(pbest_{i}^{t}|\lambda_{i}\right)$**then**$pbest_{i}^{t}=C_{i\;}^{t}$**end if****for** each j∈B(i) **do:****if**$g\left(C_{i\;}^{t}|\lambda_{j}\right)<g\left(gbest_{j}^{t}|\lambda_{j}\right)$**then**$gbest_{j}^{t}=C_{i}^{t}$**end if****end for**Remove all the vectors dominated by $C_{i}^{t}$ from PS.Add $C_{i}^{t}$ into PS if no vectors in PS dominate it.**end for****end while**


### 4.7. Model Selection

After enough iterations, there are plenty of non-dominated solutions or candidate models in the *PS*. To obtain the final refined structures, we need to assess and rank the generated models. Many methods for estimation of model accuracy have been described such as MULTICOM_CLUSTER [10], Pcons [58], PRESCO [59], DeepAccNet [60], and ProQ3D [61]. Here, for a quick ranking purpose, we use a widely used knee-based ranking method [49] in a multi-objective optimization problem to rank those models. Since the three energy functions are treated equally, AIR 2.0 has no preference to any regions of the PF. However, there will be some special solutions called ‘knee’ in the PF. In such ‘knees’ solutions, a small improvement in one objective will cause large depravation in other objectives. Thus, three objectives reach a balance that all objectives are relatively optimal and no objective can decrease further without seriously increasing other objectives. 

To obtain these ‘knee’ solutions, we adopt a utility-based method similar to the AIR 1.0, which uses the expected marginal utility to measure the importance of the solutions in the PS:

(11) $$U_{C,w}=w_{1}f_{1}\left(C\right)+w_{2}f_{2}\left(C\right)+w_{3}f_{3}\left(C\right))\;s.t.\;w_{1}+w_{2}+w_{3}=1\;and\;w_{1},w_{2},w_{3}\geq 0$$

where C is the non-dominated solution in the PS and $w_{1},w_{2},w_{3}$ are the weight coefficients. The expected margin utility is approximated by random sampling of $w_{i}$. For each conformation, we obtain a large number of utility values $\left\{U_{C,w}^{i},i=1,\ldots S\right\}$ by using different combinations of weight coefficients. The expected margin utility could be approximated by the average of these sample values:

(12) $$E\left(U_{C,w}\right)=\frac{1}{S}\sum_{i=1}^{S}U_{C,w}^{i}.$$

Then, we can rank the solutions in PS according to individual expected marginal utility and output the top-ranking solutions.

## 5. Conclusions and Future Direction

In this study, we developed a decomposition-based method AIR 2.0 for protein structure refinement. AIR 2.0 employs a decomposition strategy that divides the multi-objective optimization into a set of subproblems and optimizes them in a collaborative manner. The performance on CASP 13 refinement targets and a blind test on CASP 14 shows that AIR 2.0 is capable of achieving promising results. In the future, we will further improve the AIR refinement protocol to use deep learning methods to design new energy functions that could guide the search process and identify those local structure regions need to be refined. With this information, we could reduce the searching space largely and make the sampling process more efficient. Moreover, the new energy function could be used as the final model selection criterion to rank the models in Pareto set, which may bridge the gap between Model 1 and the best model.

## Figures and Tables

**Table 1:** Overall performance of AIR 1.0 and AIR 2.0 in terms of GDT-TS on 29 refinement targets from CASP13.

Method	Best Model (GDT-TS)	Model 1 (GDT-TS)
AIR 1.0	+1.07	+0.16
AIR 2.0	+1.98	+1.22

**Table 2:** GDT_TS comparison of AIR 2.0 and other refinement methods on CASP13 refinement targets. The results of BAKER, FEIGLAB, and Zhang come from the CASP official website. The best model among the four methods is bolded on each target.

Target	Initial Model	AIR 2.0	BAKER	FEIGLAB	Zhang
R0949	64.53	**65.12**	56.01	62.98	64.53
R0957	60.97	**64.08**	60.32	61.45	61.61
R0968s1	66.74	69.71	**78.81**	72.25	69.07
R0974s1	84.78	85.96	**99.64**	97.10	84.06
R0976D2	83.06	84.27	**89.11**	80.64	83.87
R0979	70.65	**74.18**	60.60	70.38	70.38
R0986s1	80.16	83.15	90.76	**93.21**	77.99
R0989D1	50.75	**51.12**	44.22	50.75	N/A
R0999D3	75.14	**76.94**	76.11	**76.94**	74.31
R1002D2	88.14	88.56	**89.41**	79.24	88.14
R1004D2	78.57	77.60	81.49	**93.51**	79.22
R1016	81.06	**82.11**	78.22	81.68	80.45

**Table 3:** Comparison of GDT-TS gains on different value of T.

Target	Length	T=3	T=8	T=15	T=30
R0974s1	69	−0.27	1.18	0.81	1.24
R1004D2	77	−1.05	−0.97	−0.98	−0.95
R0968s1	118	2.58	2.97	3.18	2.94
R0981D5	127	−0.79	0.59	0.44	0.20
R0959	189	3.30	3.83	3.97	3.74
R0981D3	203	0.49	0.13	0	0.13

## Data Availability

All data about our method could be obtained through the implementation above. The data about other methods is available on CASP official website.

## Availability and Implementation

AIR 2.0 is available online: www.csbio.sjtu.edu.cn/bioinf/AIR/ (accessed on 21 April 2021).
